# The Incorporation of Strontium to Improve Bone-Regeneration Ability of Mesoporous Bioactive Glasses

**DOI:** 10.3390/ma11050678

**Published:** 2018-04-26

**Authors:** Sonia Fiorilli, Giulia Molino, Carlotta Pontremoli, Giorgio Iviglia, Elisa Torre, Clara Cassinelli, Marco Morra, Chiara Vitale-Brovarone

**Affiliations:** 1 Department of Applied Science and Technology, Politecnico di Torino, Corso Duca degli Abruzzi 24, 10129 Torino, Italy; sonia.fiorilli@polito.it (S.F.); giulia.molino@polito.it (G.M.); carlotta.pontremoli@polito.it (C.P.); 2 Nobil Bio Ricerche srl, Via Valcastellana 28, 14037 Portacomaro (Asti), Italy; giviglia@nobilbio.it (G.I.); etorre@nobilbio.it (E.T.); ccassinelli@nobilbio.it (C.C.); mmorra@nobilbio.it (M.M.)

**Keywords:** mesoporous bioactive glasses, strontium, ion release, biocompatibility, osteogenesis, bone regeneration

## Abstract

Over the recent years, mesoporous bioactive glasses (MBGs) gained interest as bone regeneration systems, due to their excellent bioactivity and ability to release therapeutic molecules. In order to improve the bone regeneration ability of MBGs, the incorporation of Sr^2+^ ions, due to its recognized pro-osteogenenic potential, represents a very promising strategy. In this study, MBGs based on the SiO_2_–CaO system and containing different percentages (2 and 4 mol %) of strontium were prepared by two synthesis methods, in the form of microspheres and nanoparticles. Sr-containing MBGs were characterized by FE-SEM, XRD and N_2_ adsorption/desorption analysis. The in vitro bioactivity in SBF resulted excellent. The assessment of fibroblast cell (line L929) viability showed that Sr-containing MBGs were biocompatible both in form of micro- and nanoparticles. The osteogenic response of osteoblast-like SAOS-2 cells was investigated by analysing the expression of GAPDH, COL1a1, RANKL, SPARC, OPG and ALPL genes, as cell differentiation markers. The results indicate that the incorporation of Sr into MBG is beneficial for bone regeneration as promotes a pro-osteogenic effect, paving the way to the design of advanced devices enabled by these nanocarriers also in combination with drug release, for the treatment of bone pathologies, particularly in patients with osteoporosis.

## 1. Introduction

In the last fifty years the chemical and textural features of melt-derived bioactive glasses, discovered by Hench, have been largely modified to improve their biological performances transforming them into materials with biomedical added value [1]. 

For this reason, from the 1990s the researchers’ attention has been moved to the sol-gel synthesis approach since it provides better textural properties to the glass, such as higher surface area, porosity and homogeneity, involving also lower processing temperatures. These characteristics allow widening the range of compositions showing bioactive behaviour (up to 80 mol % of SiO_2_) and increasing the hydroxyapatite (HA) deposition rate [2,3]. The HA deposition is in fact the peculiar surface-dependent mechanism characterising bioactive materials and allowing them to form interfacial bonds with hard and soft tissues upon their reaction in physiological fluids [4,5].

From 2000’s mesoporous bioactive glasses (MBGs) have been synthesised combining the sol-gel method with the use of templating agents in order to further improve and control the textural characteristics of sol-gel bioactive glasses. Templating agents are surfactant molecules able to self-assemble in micelles, around which the hydrolysed glass precursors condense forming an ordered mesophase, whose structure depends on several factors (surfactant chemistry and concentration, temperature, pH, etc.) [2,6]. After the removal of the surfactant by calcination, an ordered mesoporous structure is obtained. Therefore, the use of these templating agents allows providing MBGs with remarkable structural features: highly ordered and tunable porosity in the range of 2 and 50 nm and higher surface area and pore volume values [7]. These peculiar characteristics increase MBG reactiveness in body fluids accelerating the process of HA deposition and making these glasses particularly suitable for bone regenerative application [8]. 

In addition to these structural characteristics, the regenerative potential of bioactive glasses is influenced by their ionic dissolution products (Si, Ca, P) [9]. In fact, it has been demonstrated that the variation of the intracellular ion concentration caused by bioactive glass dissolution leads to the activation of intracellular signalling pathways. This mechanism influences the genic expression of osteoprogenitor cells giving rise to rapid bone regeneration [10].

To stimulate a more advanced and specific cell response, therapeutic metallic ions (Cu^2+^, Sr^2+^, Ag^+^, etc.) have been recently introduced into the bioactive glass compositions [11,12,13]. These ions are able to stimulate osteogenesis, angiogenesis and to provide antibacterial properties, making them attractive as therapeutic agents in the fields of hard and soft tissue engineering [14,15]. Furthermore, the release of therapeutic ions can be synergistically combined with the delivery of pharmaceutical agents or growth factors, which can be loaded into MBG mesopores or adsorbed/grafted on the external surface of the glass particles [7].

In addition, MBGs can be exploited as multifunctional carriers in combination with a polymeric vehicle matrix for the development of novel and versatile devices for soft and hard tissue regeneration. With this purpose, the incorporation of Cu-containing MBGs into a thermosensitive hydrogel to obtain a non-invasive injectable formulation for prolonged and localized therapeutic ion release has been recently reported by the authors [16]. Another intriguing application foresees their incorporation in a Type I collagen matrix to produce bone like, hybrid materials to promote the natural bone remodeling in osteoporotic tissues, as recently reported by Montalbano et al. [17].

Among the elements with potential therapeutic properties, strontium has received remarkable interest, due to its well-known role in bone metabolism [18,19,20,21,22] by exercising anabolic and anti-catabolic effects. Indeed, due to its chemical analogy to calcium, strontium is able to exploit the calcium sensing receptors present on both osteoblastic and osteoclastic cells to activate their downstream signalling pathways, leading to the promotion of osteoblast replication, differentiation and survival as well as downregulating osteoclast activities [23]. Furthermore strontium is normally present in the hydroxyapatite crystals of human bones due to its ability of exchange with calcium in the HA reticulum [14]. 

Several studies conducted on bioactive glasses where calcium has been partially substituted by strontium have shown its beneficial effect in promoting bone remodelling. In particular, Moghanian et al. [24] tested the response of mouse osteoblast-like cells on solid discs composed of Sr-containing 58S glass (60% SiO_2_–36% CaO–4% P_2_O_5_ mol %) and evidenced that the presence of strontium was able to increase the metabolic behaviour of osteoblasts and to promote their proliferation and the alkaline phosphatase (ALP) activity. Enhanced osteoblastic activity was also observed by Taherkhani et al. [25], who exposed human osteosarcoma cells to culture media treated with ionic dissolution products of the same type of glass. More recently, Naruphontjirakul et al. [12] investigated murine pre-osteoblast cell response to silica nanoparticles obtained by the Stöber method and subsequently enriched with calcium and strontium, as well as the cell response to their ionic dissolution products, and reported an enhanced ALP activity and osteogenic differentiation in presence of nanoparticles containing strontium. 

In this work, different molar concentrations of strontium have been incorporated into the framework of a mesoporous glass with SiO_2_–CaO composition. Sr-containing MBGs have been synthesised by two different approaches, a batch sol-gel procedure and an aerosol-assisted spray-drying method, to produce nano- and micro-sized spheres, respectively. Besides the size of the final particles, the two preparation methods allowed to obtain Sr-substituted samples with different structural features in term of specific surface area, pore volume and pore size and, consequently, to investigate how these aspects may influence the bioactivity and the biological response in terms of cytocompatibility and pro-osteogenic effect. 

## 2. Materials and Methods 

### 2.1. Preparation of Sr-Containing MBGs (Sr-MBGs)

MBGs containing different amount of Sr (2 and 4 mol %) were synthesised through two different synthesis approaches in order to obtain particles with different size and structural parameters (i.e., specific surface area, pore size).

#### 2.1.1. Preparation of Sr-Containing MBG Samples by Aerosol-Assisted Spray Drying Method

Based on a modification of the procedure reported by Pontiroli et al. for a binary SiO_2_-CaO composition [26], MBG micro-particles with different molar percentage of Sr (molar ratio Sr/Ca/Si = 2/13/85 and Sr/Ca/Si = 4/11/85, hereafter named as MBG_Sr2%_SD and MBG_Sr4%_SD respectively) were synthesised by aerosol-assisted spray drying method. Briefly, 2.0 g of the non-ionic block copolymer Pluronic P123 (EO_20_PO_70_EO_20_, average M_n_ ~5800, Sigma Aldrich, Milan, Italy) were dissolved in 85.0 g of double distilled H_2_O (ddH_2_O) (solution A). In a separate batch, 10.7 g of TEOS were pre-hydrolysed under acidic conditions using 5.0 g of an aqueous HCl solution at pH = 2 until a transparent solution was obtained (solution B). Solution B was then added drop by drop into solution A and kept stirring for 1 h. Depending on the glass composition, the proper amounts of strontium chloride hexahydrate (SrCl_2_·6H_2_O, for analysis EMSURE^®^ ACS) and calcium nitrate tetrahydrate (Ca(NO_3_)_2_·4H_2_O, 99%, Sigma Aldrich, Milan, Italy), reported in Table 1, were added.

The final solution was stirred for 15 min and then sprayed (Büchi, Mini Spray-Dryer B-290, Büchi Labortechnik AG, Flawil, Switzerland) using nitrogen as the atomizing gas with the following parameters: inlet temperature 220 °C, N_2_ pressure 60 mmHg and feed rate 5 mL/min. The obtained powder was calcined at 600 °C in air for 5 h at a heating rate of 1 °C min^−1^ using a furnace (Carbolite 1300 CWF 15/5, Carbolite Ltd., Hope Valley, UK), in order to remove the template agent. 

#### 2.1.2. Preparation of Sr-Containing MBG Samples by Sol-Gel Synthesis (Base-Catalysed)

MBG nanoparticles containing different amount of Sr (2 mol % of Sr, molar ratio Sr/Ca/Si = 2/13/85, and 4 mol % of Sr, molar ratio Sr/Ca/Si = 4/11/85, named hereafter as MBG_Sr2%_SG and MBG_Sr4%_SG respectively) were synthesised by a base-catalysed sol-gel synthesis, based on a modified procedure reported by the authors [16]. In particular, 6.6 g cetyltrimethylammonium bromide (CTAB ≥98%, Sigma Aldrich, Milan, Italy) and 12 mL NH_4_OH (Ammonium hydroxide solution, Sigma Aldrich, Milan, Italy) were dissolved in 600 mL of ddH_2_O under stirring for 30 min. Then, 30 mL of tetraethyl orthosilicate (TEOS, Tetraethyl orthosilicate, Sigma Aldrich, Milan, Italy), calcium nitrate tetrahydrate (Ca(NO_3_)_2_·4H_2_O, 99%, Sigma Aldrich, Milan, Italy) and strontium chloride hexahydrate (SrCl_2_·6H_2_O, for analysis EMSURE^®^ ACS) as reported in Table 2, were added and kept under vigorous stirring for 3 h. 

The powder was collected by centrifugation (Hermle Labortechnik Z326, Hermle LaborTechnik GmbH, Wehingen, Germany) at 10,000 rpm for 5 min, washed once with distilled water and two times with absolute ethanol. The final precipitate was dried at 70 °C for 12 h and then calcined at 600 °C in air for 5 h at a heating rate of 1 °C min^−1^ using a furnace (Carbolite 1300 CWF 15/5 Carbolite Ltd., Hope Valley, UK), in order to remove CTAB. 

### 2.2. Characterization of Sr-MBGs 

Wide-angle (2ϑ within 15–80°) X-ray diffraction measurements (X’Pert PRO, PANalytical, Almelo, The Netherlands) were performed using CuKα radiation at 40 kV and 40 mA. 

MBG_Sr_SD were dispersed on a conductive carbon tape prior to SEM observation (Phenom XL, Phenom-World, Eindhoven, The Netherlands). The MBG_Sr_SG particles morphology was analysed by Field-Emission Scanning Electron Microscopy (FE-SEM) using a ZEISS MERLIN instrument (Oberkochen, Germany). In particular, 10 mg of MBG_Sr_SG powders were dispersed in 10 mL of isopropanol by ultrasonication using an ultrasonic bath (Digitec DT 103H, Bandelin, Berlin, Germany) for 5 min to obtain a stable suspension. A drop of the obtained suspension was deposited on a carbon-coated copper grid (3.05 mm Diam.200 MESH, TAAB, Aldermaston, Berks, UK) and allowed to dry. A Cr layer was deposited on powders before SEM and FE-SEM analyses in order to enhance the sample conductivity.

Nitrogen adsorption/desorption isotherms were measured (ASAP2020, Micromeritics ASAP 2020 Plus Physisorption, Norcross, GA, USA), at the temperature of −196 °C. Before nitrogen adsorption–desorption measurements, each sample was degassed at 150 °C for 3 h. The Brumauer–Emmett–Teller (BET) model was applied to determine the specific surface areas (SSA_BET_) of the samples. The pore size distribution was calculated through the DFT method (Density Functional Theory) using the NLDFT kernel of equilibrium isotherms (desorption branch).

### 2.3. Sr^2+^ Ions Release Tests

In order to evaluate the concentration of released Sr^2+^ ions, the powders were soaked in Tris HCl buffer (Tris(hydroxymethyl)aminomethane (Trizma) (Sigma Aldrich, Milan, Italy) 0.1 M, pH 7.4) at concentration of 250 μg/mL, according to the protocol described by Shi et al. [27]. In particular, 5 mg of powder were suspended in 20 mL of buffer up to 14 days at 37 °C in an orbital shaker (Excella E24, Eppendorf) with an agitation rate of 150 rpm. At defined time points (3 h, 24 h, 3 days, 7 days and 14 days) half of the supernatant was collected after centrifugation at 10,000 rpm for 5 min (Hermle Labortechnik Z326, Wehingen, Germany) and replaced by the same volume of fresh buffer solution to keep constant the volume of the release medium. The release experiments were carried out in triplicate.

The concentration of Sr ions was measured by Inductively Coupled Plasma Atomic Emission Spectrometry Technique (ICP-AES) (ICP-MS, Thermoscientific, Waltham, MA, USA, ICAP Q), after appropriate dilutions. In order to assess the initial amount of strontium incorporated during the synthesis all samples were dissolved in a mixture of nitric and hydrofluoric acids (0.5 mL of HNO_3_ and 2 mL of HF for 10 mg of powder) and the resulting solutions were analysed via ICP analysis.

### 2.4. In Vitro Bioactivity of Sr-Containing MBGs

*In vitro* bioactivity test was performed to evaluate the apatite-forming ability of Sr-MBGs in simulated body fluid (SBF). To this aim, 30 mg of Sr-MBGs were soaked in 30 mL of SBF, according to literature [28]. The samples were kept immersed at 37 °C up to 14 days in an orbital shaker (Excella E24, Eppendorf, Milan, Italy) with an agitation rate of 150 rpm. At each time point (3 h, 1 day, 3 days, 7 days and 14 days), the suspension was centrifuged at 5000 rpm for 5 min, in order to separate the powder from the solution. The pH of each recovered supernatant was measured, and the powder was washed with distilled water and dried in oven at 70 °C for 12 h prior FE-SEM and XRD analysis to evaluate the apatite layer formation. 

### 2.5. In Vitro Biological Assessment of Sr-Containing MBGs

The biological response to MBG_Sr2%_SD and MBG_Sr2%_SG and to their ionic release products was assessed by following two different experimental approaches. In particular, through a direct contact method, where cells were seeded directly on the MBG particles, and through a not-contact method, according to which the Sr-MBG suspensions were placed in a Transwell^®^ membrane insert (<3 µm pore, SARSTEDT AG & Co., Numbrecht, Germany) to allow the passage of the particle dissolution products.

#### 2.5.1. Inflammatory Response of Sr-Containing MBGs

The inflammatory response test was conducted in direct contact mode, using cells and Sr-MBG particles at concentration of 1 mg/mL. For these tests the murine macrophage cell line J774a.1 (European Collection of Cell Cultures) was used. Before the tests, cells were maintained in Dulbecco’s modified Eagle’s medium (Gibco Invitrogen, Cergy-Pontoise, France) supplemented with 10% fetal bovine serum, penicillin (100 U·mL^−1^), streptomycin (100 μg·mL^−1^) and 4 mM l-glutamine. Cells were grown in a 100% humidified incubator at 37 °C with 10% CO_2_ and passaged 2–3 days before use. Then the J774a.1 cells (2 × 10^4^ mL^−1^) were seeded onto 24-well tissue culture polystyrene plates (Falcon™), containing the Sr-MBG particles. After 4 h, the RNA from J774.a1 cells was isolated by using the Maxwell^®^ RSC simply RNA Cells Kit (Promega Italia s.r.l, Milan, Italy) and reverse transcribed by the High-Capacity cDNA Reverse Transcription Kit (Applied Biosystems, Foster City, CA, USA). Real-time PCR was performed through the Applied Biosystems StepOne Plus instrument with 2.2 Step-one software version. Mouse interleukin-1β (IL-1β), interleukin-6 (IL-6), tumour necrosis factor alpha (TNFα) and Tyrosine 3-Monooxygenase/Tryptophan 5-Monooxygenase Activation Protein Zeta (YWHAZ) were chosen from the collection of the TaqMan Gene Expression Assays as primer sets (Applied Biosystems Assay’s ID: Mm01336189_m1, Mm99999062_m1, Mm00443258_m1, Mm03950126_s1 respectively). Real time PCR was performed in duplicate for all samples in a volume of 20 µL and, after an initial denaturation at 95 °C for 10 min, the PCR amplification was run for 40 cycles at 95 °C for 15 s and at 60 °C for 1 min. The content of cDNA samples was normalized through the comparative threshold cycle (ΔΔCt) method, consisting in the normalization of the number of target gene copies versus the endogenous reference gene YWHAZ. 

#### 2.5.2. Biocompatibility Test of Sr-Containing MBGs

Fibroblast cell line L929 was used to assess the biocompatibility of Sr-MBGs. Experimental cell culture medium (BIOCHROM KG, Berlin, Germany), composed by Minimum Eagle’s Medium without L-glutamine, 10% fetal bovine serum, streptomycin (100 g/L), penicillin (100 U/mL), and 2 mmol/L L-glutamine, was placed in 250 mL plastic culture flask (Corning TM, Corning, NY, USA). Cells were cultured at 37 °C in a humidified incubator equilibrated with 5% CO_2_. Cells were harvested prior to confluence by means of a sterile trypsin-EDTA solution (0.5 g/L trypsin, 0.2 g/L EDTA in normal phosphate buffered saline, pH 7.4), re-suspended in the experimental cell culture medium and diluted to 1 × 10^5^ cells/mL.

A preliminary qualitative assessment was carried out through optical imaging of the cells in direct contact with Sr-MBGs particles. In parallel, cell viability tests were performed in Transwell^®^ permeable inserts. Briefly, fibroblast cells were seeded on polystyrene plate below the Transwell^®^ insert containing 1 mg/mL of Sr-MBG suspension, and after 72 h of incubation cell viability was evaluated through MTT assay. This assay allows assessing the possible toxic effect of particle dissolution products on cells, by evaluating the reduction of the mitochondrial succinate dehydrogenase (SDH) enzyme activity, normally involved in the citric acid cycle. For the execution of the MTT test, cells were incubated with a 1 mg/mL solution of soluble tetrazolium salt (3-(4,5-dimethylthiazol–2yl)-2,5 diphenyl tetrazolium bromide). During the subsequent two hours of incubation at 37 °C, the succinate dehydrogenase enzyme causes the transformation of tetrazolium salts into a yellow soluble substance first and then into a blue water-insoluble product, the formazan precipitate. From the quantification of the precipitate product is possible to evaluate the degree of the enzyme activity and, consequently, the number of metabolically active cells. To perform this evaluation, the formazan precipitate was dissolved with dimethylsulphoxide and was spectrophotometrically measured at a wavelength of 570 nm, providing an optical density (OD) value. Cells grown on polystyrene plate were used as negative control, while cells grown with the addition of 20 µL of a solution of 0.08 mg/mL of Sodium nitroprusside (NPS) were used as the positive one. 

#### 2.5.3. Osteogenic Response to Sr-Containing MBGs 

Osteoblast-like SAOS-2 cells were cultured at 37 °C in a humidified incubator equilibrated with 5% CO_2_. Cell suspension was obtained by adding 2 mL of a sterile 0.5% Trypsin-EDTA solution (GIBCO by Life Technology, ref 15400-054, Thermoscientific, Waltham, MA, USA) to a 250 mL cell culture flask (Corning™), re-suspended in the experimental cell culture medium and diluted to 1.45 × 10^5^ cells/mL. 

For these experiments, 5 mL of the cell suspension were seeded onto 24-well tissue culture polystyrene plates (Falcon™), provided with the Transwell^®^ insert containing 1 mg/mL of Sr-MBG suspension. After 72 h and 7 days of incubation, the expression of GAPDH, COL1a1, RANKL, SPARC, OPG and ALPL genes as cell differentiation markers was assessed using the real time reverse transcription polymerase chain reaction (qRT-PCR) (Applied Biosystems Assay’s ID: Hs00266705_g1, Hs00164004_m1, Hs00234160_m1, Hs00243519_m1, Hs00900358_m1, Hs01029144_m1, respectively). 

The RNA from SAOS-2 cells was isolated by using the Maxwell^®^ RSC simply RNA Cells Kit (Promega), by following the manufacturer’s instructions. RNA was reverse transcribed by the High-Capacity cDNA Reverse Transcription Kit (Applied Biosystems) and RNA quantitation was performed before starting the Rt-PCR using Quantifluor system kit (Promega).

Real-time PCR was performed in the Applied Biosystems StepOne Plus instrument (Applied Biosystems). The content of cDNA samples was normalized by making use of the comparative threshold cycle (ΔΔCt) method.

### 2.6. Statistical Analysis

Experimental data are reported as mean ± standard deviation. Statistical differences between groups were analysed using two-way ANOVA using Tukey’s post-hoc test and one-way ANOVA using Tukey’s pairwise post-hoc test. Statistical significance was represented as * *p* < 0.05, ** *p* < 0.01 and *** *p* < 0.001.

## 3. Results and Discussion

### 3.1. Characterization of Sr-Containing MBGs 

#### 3.1.1. Morphological and Structural Characterization 

**Figure 1:**
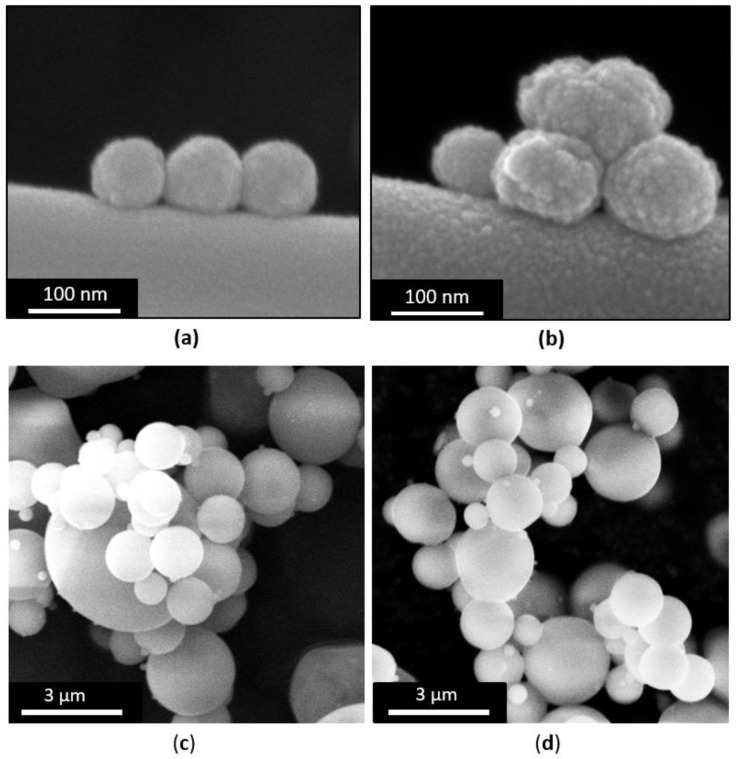
FE-SEM images of (**a**) MBG_Sr2%_SG; (**b**) MBG_Sr4%_SG; (**c**) MBG_Sr2%_SD; (**d**) MBG_Sr4%_SD.

FE-SEM images (Figure 1a,b) of MBG_Sr2%_SG and MBG_Sr4%_SG revealed particles with uniform spherical morphology and size ranging between 100 and 200 nm. At variance, spray-dried samples (Figure 1c,d) were spherical particles with micrometric size between 0.5 and 5 µm, in analogy with those obtained in previous works [26,29]. Morphological analysis evidenced that for both methods the introduction of higher amount of strontium precursor did not lead to significant morphological variations of the final particles. 

**Figure 2:**
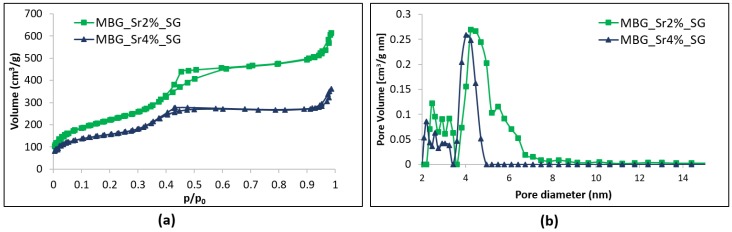
N_2_ adsorption-desorption curves of MBG_Sr2%_SG and MBG_Sr4%_SG: (**a**) isotherms; (**b**) DFT pore size distributions.

Nitrogen adsorption-desorption isotherms (Figure 2a) of MBG_Sr2%_SG and MBG_Sr4%_SG samples are both classified as type IV isotherms, conventionally associated with mesoporous materials, with H2 hysteresis loop. Pore size distributions of both samples showed uniform mesopores with a mean diameter of about 4 nm (Figure 2b). The increase of Sr molar concentration from 2 to 4 mol % led to a decrease in the specific surface area (SSA) from 803 to 551 m^2^·g^−1^ and of the pore volume from 0.82 to 0.45 cm^3^·g^−1^ [18]. In the literature, other works have shown that the incorporation of increasing amount of strontium, or trivalent element as Ce, into the framework of MBGs, affected their structural features [18,22,30], leading to a reduction of specific surface area and pore volume, associated to the appearance of disorganized non-porous domains [30].

Most likely, the higher incorporation of Sr^2+^ may interfere during the self-assembly interactions between SiO_4_^4−^ species and the cationic heads of the template, which can cause structural defects in the polymerized framework and the consequent alteration of the final mesopore structure [18].

**Figure 3:**
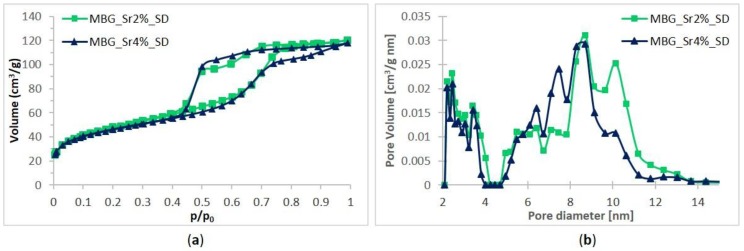
N_2_ adsorption-desorption curves of MBG_Sr2%_SD and MBG_Sr4%_SD: (**a**) isotherms; (**b**) DFT pore size distributions.

Spray-dried samples also showed IV type isotherms confirming their mesoporous nature, with a pronounced H2 hysteresis (Figure 3a). According to the literature, this could be associated to the presence of a complex pore network undergoing the pore-blocking phenomenon, due to the presence of mesopores connected to the external surface through narrow necks that caused the empting of pores at lower vapour pressures [31,32]. This less organized porous structure could result from the fast kinetics of the spray-drying process, which reduce the time available for the mesophase organization and the degree of silica framework condensation. The values of specific surface area, pore volume and average pore size are reported in Table 3. The obtained spray-dried MBGs showed SSA values comparable to those previously obtained by our research group through analogous synthesis without Sr incorporation [26], evidencing the reliability and versatility of this preparation method. 

A slight decrease of SSA values, from 167 m²·g^−1^ of MBG_Sr2%_SD to 154 m²·g^−1^ of MBG_Sr4%_SD, was observed for increasing amount of substituting element, as already reported for Sr-containing glasses [33]. Nevertheless, the obtained SSA values are remarkably higher than those of conventional sol-gel glasses, allowing an improved reactivity in physiologic environment [8]. As expected, based on the features of the used templating agent (Pluronic P123), spray-dried samples showed an average pore size of about 8 nm [34], irrespective of the amount of strontium in the synthesis solution. 

#### 3.1.2. Strontium Ion Release from Sr-Containing MBGs

Release tests carried out in Tris HCl medium evidenced, as expected, that the released ionic concentration (ppm) of Sr^2+^ ions is dependent on MBG composition, as higher Sr substitution resulted in higher released amount. Analogous behavior was reported for similar systems, such as Sr-containing bioactive glass nanoparticles or Sr-substituted MBG scaffolds, which showed a release capacity well-correlated to the Sr^2+^ incorporated amounts [12,21,24,35].

The release profile of Sr^2+^ from MBG_Sr2%_SG and MBG_Sr4%_SG showed a burst effect in the first 3 h for both samples followed by a plateau (as reported in Table 4). The fast ion release kinetics is attributed to the remarkably high surface area and to the particle size (short diffusion paths), which allow fast ion diffusion inside the porous structure [16]. The chemical composition of MBG_Sr2%_SG and MBG_Sr4%_SG, determined by ICP-AES, revealed a reduced incorporation of strontium compared to the nominal values. A similar behavior was reported for Sr-substituted glasses, prepared by sol-gel method, which revealed incorporated amounts lower than the nominal ones [36]. This difference can be tentatively ascribed to the larger ionic radius of strontium (1.16 Å) compared to calcium (0.94 Å) [33], which can limit its incorporation into the framework during silica polymerization. Moreover, a lower charge density (charge/ionic radius) can account for weaker charge interactions between Sr^2+^ ions and the silicate framework and can contribute to the observed lower Sr^2+^ incorporation rate.

However, as far as MBG_Sr4%_SG sample is concerned, the total amount of incorporated strontium was released with a final concentration of about 7.6 ppm. Similarly, the final released concentration of Sr^2+^ from MBG_Sr2%_SG results slightly lower compared to that expected based on the incorporated amount (about 95%), with a final concentration of about 2.4 ppm (Table 4).

Spray-dried MBGs showed also a burst release of Sr^2+^ in the first 3 h of soaking in Tris-HCl, followed by a stationary trend. Similarly, the fast ion release could be attributed to the high accessibility of medium into the porous structure and the fast ionic exchange reactions occurring at the surface. The final strontium concentration after 72 h of soaking was 7.2 ppm for MBG_Sr2%_SD and 13.2 ppm for MBG_Sr4%_SD, which represented respectively the 94% and the 100% of the amount initially incorporated into the two samples, which resulted very similar to the theoretical value (see Table 4). 

#### 3.1.3. In Vitro Bioactivity of Sr-Containing MBGs

**Figure 4:**
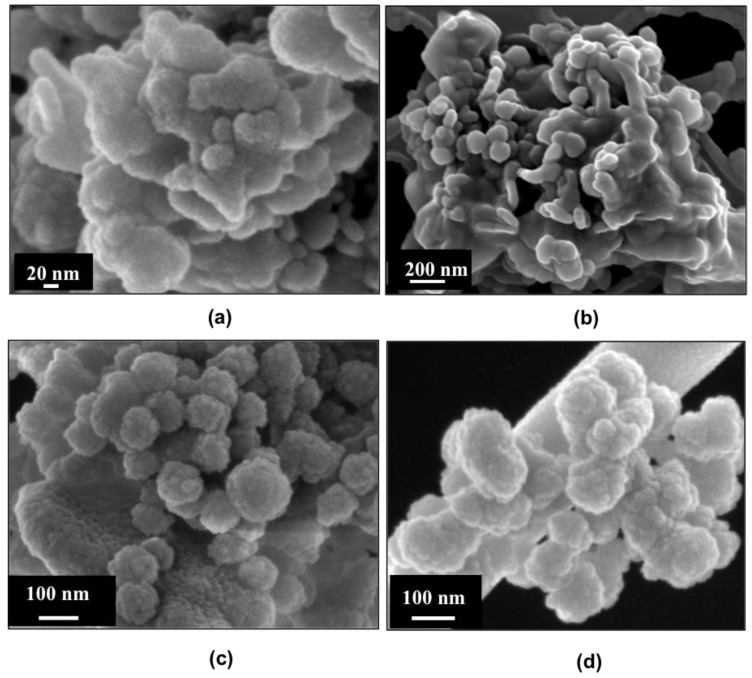
FESEM images of powders after soaking in SBF: (**a**) MBG_Sr2%_SG after 3 days; (**b**) MBG_Sr2%_SG after 7 days; (**c**) MBG_Sr4%_SG after 3 days; (**d**) MBG_Sr4%_SG after 7 days.

The very high SSA and the mesoporous texture of MBGs enhance their bioactivity, namely the ability to induce the formation of a HA layer on their surface through a series of ionic exchanges with physiologic fluids [37]. In fact, while melt-derived and non-porous sol-gel glasses exhibit bioactivity after several days of soaking in SBF, MBGs are able to induce the deposition of the HA layer in shorter time [8]. This behaviour was also observed for the Sr-containing samples synthetized in this work. In particular, MBG_Sr2%_SG and MBG_Sr4%_SG showed the formation of nanometric crystals of HA after 3 days of soaking in SBF that grew in size during the test, causing the embedding of MBG particles after 7 days (Figure 4).

**Figure 5:**
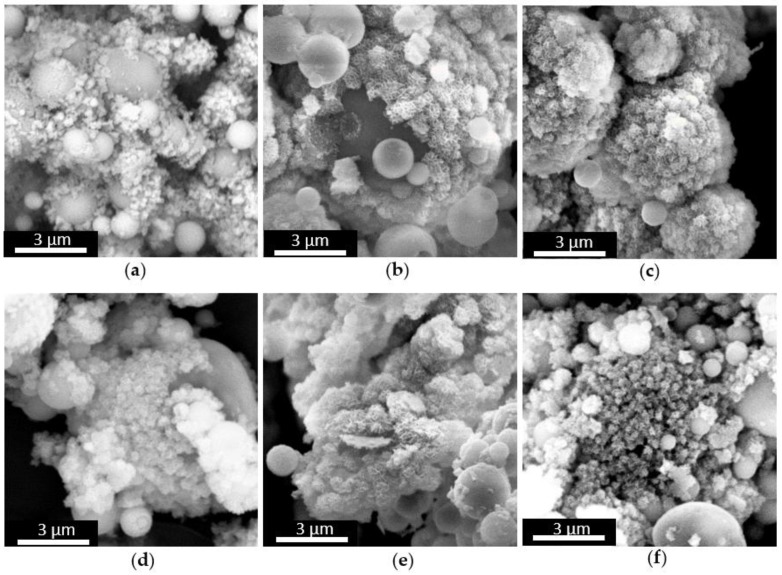
FESEM images and XRD spectra of MBG_Sr_SD after different time of soaking in SBF: (**a**) MBG_Sr2%_SD after 1 day; (**b**) MBG_Sr2%_SD after 3 days; (**c**) MBG_Sr2%_SD after 7 days; (**d**) MBG_Sr4%_SD after 1 day; (**e**) MBG_Sr4%_SD after 3 days; (**f**) MBG_Sr4%_SD after 7 days.

Spray-dried MBGs, irrespective of the strontium content, exhibited a faster bioactive behaviour inducing the deposition of numerous HA crystals on their surface after only 1 day of soaking in SBF (Figure 5). After 7 days of soaking, the particles appeared completely covered by a compact layer of HA having the typical cauliflower morphology.

**Figure 6:**
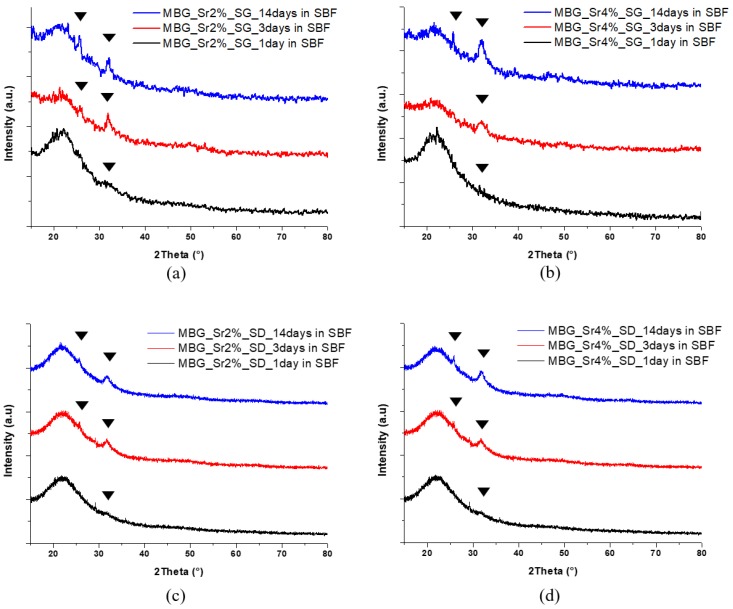
XRD spectra after different time of soaking in SBF of: (**a**) MBG_Sr2%_SG; (**b**) MBG_Sr4%_SG; (**c**) MBG_Sr2%_SD; (**d**) MBG_Sr4%_SD.

The precipitation of a HA layer on the Sr-containing samples and its crystalline nature was confirmed by XRD analysis: new peaks appeared at 25.8 and 32.0 2θ degrees in all MBG samples matching the HA reference (00-001-1008) as can be observed in Figure 6. 

The effect of strontium content in bioactive glasses on HA formation remains an open issue in the literature. Arepalli et al. [38] and Taherkhani et al. [25] reported that the introduction of relatively high amount of strontium increased the HA formation both for melt-derived and sol-gel bioactive glasses, due to the decrease of the silica network connectivity. On the contrary, Moghanian et al. [24] and Hu et al. [39] found that the partial molar substitution of Ca^2+^ with Sr^2+^ induced a decreased release of calcium ions from the glass into the SBF, causing consequently the reduction of its apatite-forming ability. In this work, based on morphological and structural analysis, the kinetics of HA formation of Sr-containing MBGs appeared not significantly influenced by the amount of incorporated strontium, compared to un-substituted samples.

Furthermore, during the in vitro bioactivity test the pH of the SBF solution remained below 7.8 which is considered the threshold value for allowing osteoblasts to maintain their physiological activity [40], making these systems appropriate for bone regenerative applications.

The overall characterization demonstrated the versatility and the reliability of the two employed preparation methods to produce Sr-containing MBGs with tailored morphology (particle size), structural features (surface area, pore size), incorporated amount of bivalent ions and release properties, without affecting the excellent bioactive behaviour typical of MBGs. 

### 3.2. In Vitro Biological Assessment of Sr-Containing MBGs

The following in vitro biological assessment aimed to evaluate if the developed Sr-containing samples and their released products allow cell survival and can exhibit a therapeutic effect by stimulating pre-osteoblastic cells. In order to study this aspect and the role of both the particles size and the structural features on the induced biological response, the experiments described in Section 2.5, were carried out on MBG_Sr2%_SD and MBG_Sr2%_SG.

#### 3.2.1. Biocompatibility of Sr-Containing MBGs

**Figure 7:**
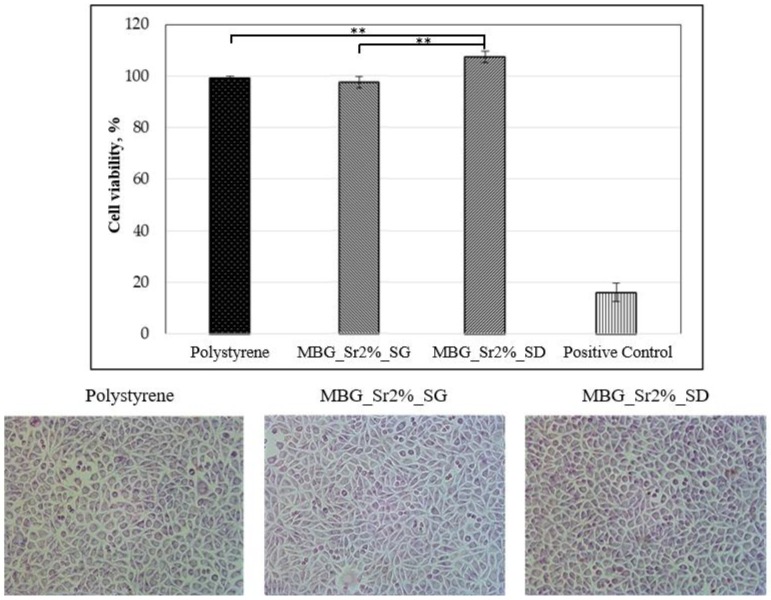
Section above: quantification of cell viability through MTT assay for MBG_Sr2%_ SG and MBG_ Sr2%_SD compared with polystyrene (negative control) and positive control (polystyrene with 0.08 µg/mL of NPS). (** *p* ≤ 0.01). Section below: optical images of cells after 72 h of incubation with MBG_Sr2%_ SG and MBG_ Sr2%_SD at concentration of 1 mg/1 mL, compared to cells seeded on polystyrene plate.

Fibroblast cells were selected as a model cell line to explore the biocompatibility of MBG_Sr2%_SG and MBG_Sr2%_SD samples, by using a polystyrene plate as negative control and a polystyrene plate added with 0.08 mg/mL of Sodium Nitroprusside to induce cell death as positive control. The results of cell viability tests and the optical images of cell morphology are reported in Figure 7. Overall, it is evident that the presence of Sr-containing MBG particles did not significantly alter the cell morphology, which appeared comparable to that shown by the cells grown on polystyrene plate (negative control). Results from MTT assay evidenced that both MBG_Sr2%_SG and MBG_Sr2% SD showed excellent biocompatibility, since the obtained cell viability percentages were far higher than 70%, which according to the international standard ISO 10993-5: 2009-Biological Evaluation of Medical Devices Tests for In vitro cytotoxicity-, is the required minimum value for considering biocompatible the tested material. In particular, the biocompatibility of MBG_ Sr2%_SD is remarkably high as it seems to promote cell proliferation, showing even higher cell viability compared to the negative control. 

#### 3.2.2. Inflammatory Response of Sr-Containing MBGs

Usually, one of the major issues associated to nano- and micro-sized material is the induction of the inflammatory response, which could be even more severe if associated to particle dissolution products. In order to investigate the effect of both material size and ion release from the particles in inducing pro-inflammatory reactions, murine macrophage cell line were seeded in the presence of MBG_Sr2%_SG and MBG_Sr2%_SD and the expression of three pro-inflammatory genes was evaluated. 

In particular, the following genes were considered: interleukin 1β (IL1β), an important mediator of the inflammatory response and involved in a variety of cellular activities, including cell proliferation, differentiation and apoptosis; interleukin 6 (IL6), an important mediator of fever and of the acute phase response and, finally, tumour necrosis factor-α (TNF-α), involved in the systemic inflammation response and in the make up of the acute phase reaction. 

**Figure 8:**
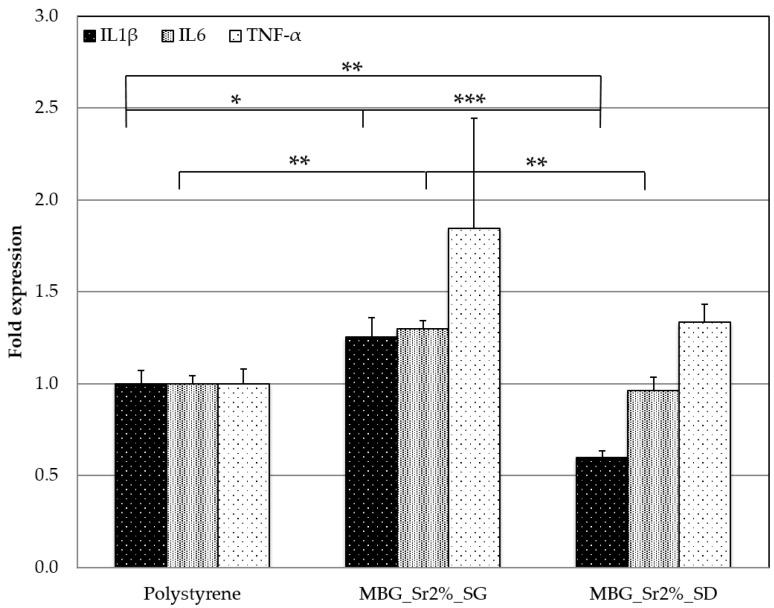
Gene expression of macrophages after 4 h of cell culture in direct contact with MBG_Sr2%_SD and MBG_Sr2%_SG. Fold expression of cytokines IL1β, IL 6 and TNFα. (* *p* < 0.05, ** *p* < 0.01 and *** *p* < 0.001).

Figure 8 shows the fold expression of the listed genes obtained for MBG_Sr2%_SD and MBG_Sr2%_SG compared with the polystyrene plate control. The results showed that there was no increase in the inflammatory response due to the presence of both Sr-containing MBGs. In particular, a significant reduction of IL6 and of IL1β expression was detected for MBG_Sr2%_SD compared with polystyrene and MBG_Sr2%_SG. At variance, a slightly increase of cytokines expression was identified for the MBG_Sr2%_SG. The reason could be associated to the smaller size of the latter compared to the micro-size of MBG_Sr2%_SD. Several studies have shown that nanoparticles and sub-micro particles are more pro-inflammatory than their microparticle counterparts [41,42,43] although the question is still controversial as conflicting results have been obtained in other studies [44,45]. In particular, Kusaka et al. reported a study on the effect of amorphous silica particle size on macrophages inflammatory response, where they showed an increase in the expression of IL1β and consequently of TNFα for particles with a size between 30 nm–1000 nm due to a lysosomal damage caused by their internalization by macrophages [42]. 

On the other hand, the reduction of IL1β, IL6 and TNFα expression found for the MBG_Sr2%_SD, could be also associated to the release of strontium ions. Indeed, in the literature, the release of Sr^2+^ is reported to produce a positive effect on cytokines production, decreasing the inflammatory reaction. Renaudin et al. demonstrated that monocyte cells, stimulated with lipopolysaccharide (LPS) to induce an inflammatory reaction, produced a significantly lower amount of TNFα when incubated with particles able to release strontium ions [46]. Since MBG_Sr2%_SD was able to release three times the amount of Sr^2+^ compared to MBG_Sr2%_SG (see Section 3.1.2), it is possible to postulate a correlation between the amount of released Sr^2+^ and the reduction of pro-inflammatory cytokines.

#### 3.2.3. Pro-Osteogenesis of Sr-Containing MBGs 

**Figure 9:**
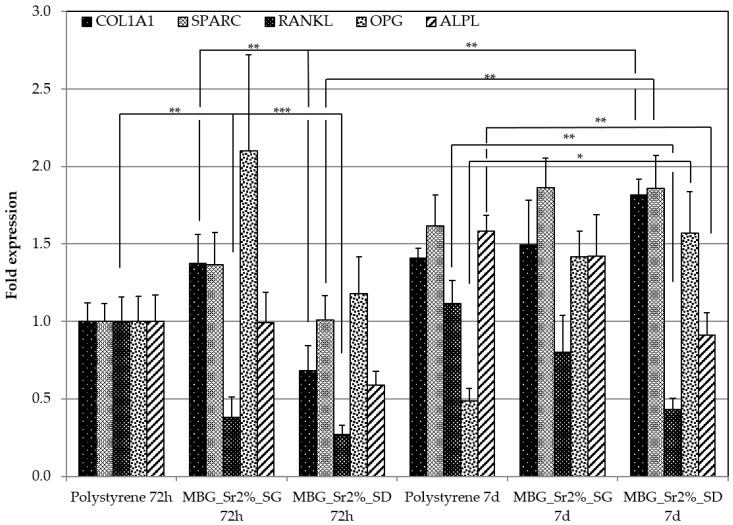
The expression level of the COL1A1, SPARC, RANKL, OPG and ALPL genes of osteoblast-like cells (SAOS2) analyzed by qRT-PCR at 72 h and at 7 days (* *p* ≤ 0.05, ** *p* ≤ 0.01 and *** *p* ≤ 0.001).

Pro-osteogenic response of Sr-containing MBGs, was evaluated by performing the experiments with Transwell^®^ permeable insert, which by hindering to a large extent the direct contact between the particles and the cells, allowed to elucidate the role of the released ions. The effect on the expression of pro-osteogenic genes due to the ionic extracts of MBG_Sr2%_SG and MBG_Sr2%_SD was evaluated by Rt-PCR analysis and the related results are shown in Figure 9.

The pro-osteogenic effect was evaluated by considering the expression of different type of genes involved in the process of bone regeneration. In particular, RANKL and OPG genes are fundamental markers for the assessment of the osteogenic potential. In fact, OPG can reduce the production of osteoclasts and consequently the excessive bone resorption, by inhibiting the differentiation of osteoclast precursors. When OPG binds to RANKL on osteoblasts, it blocks the RANKL-RANK interaction between osteoblasts and osteoclast precursors, then inhibiting their differentiation into mature osteoclasts [47,48]. Therefore, the RANKL/OPG ratio is widely considered an important indication of bone mass and skeletal integrity [49,50].

At 72 h and 7 days, gene expression analysis showed a decreased expression of RANKL for both types of Sr-containing MBGs, with a RANKL/OPG ratio in favour of OPG expression being ≤1 (see Table 5). Interestingly, at 7 days this ratio switched in favour of RANKL in the polystyrene cultured cells, while remained almost unchanged in presence of both Sr-containing MBGs. In particular, this ratio was 2.291 for cells grown on polystyrene plates, and 0.563 and 0.275 in the presence of MBG_Sr2%_SG and MBG_Sr2%_SD, respectively. The increase of the OPG expression of cells cultured in the presence of ionic extracts from Sr-containing MBGs suggests the inhibition of osteoclastogenesis, confirming the osteogenic activity induced by released Sr^2+^ ions in amount suitable to exert in vitro a therapeutic effect.

A non-negligible increase of the RANKL/OPG ratio was observed for MBG_Sr2%_SG at 7 days. The increased expression of RANKL gene of osteoblast cells is consistent with the results of pro-inflammatory response experiment, in which MBG_Sr2%_SG showed an increased expression of TNFα gene. Indeed, in inflammatory bone diseases, the systemic increase of TNF stimulates the generation of osteoclast precursors (OCPs) in the bone marrow and enhances their transfer into the bloodstream. These OCPs differentiate into osteoclasts and sustain their production in response to TNF and RANKL, by significantly affecting the final bone volume and turnover [51]. However, the RANKL increase of cells cultured with MBG_Sr2%_SG was not enough substantial to overturn the ratio. An over-expression of COLL1A1 gene (an early precursor of mineralization) was detected for MBG_Sr2%_SG at 72 h, without further increase at 7 days. Conversely, an initial down regulation was detected for MBG_Sr2%_SD, which at variance showed a significant over expression at 7 days.

In addition, we found an earlier and late expression of osteonectin in presence of both MBG_Sr2%_SG and MBG_Sr2%_SD particles. Osteonectin, known as SPARC, is a glycoprotein expressed in bone during the remodelling process. The over- expression of SPARC at 72 h and in particular after 7 days, may suggest that strontium ions could have an active role during bone regeneration. Osteonectin is synthesized by osteoblast cells and plays a role in binding bone minerals (HA) and type I collagen [52,53]; furthermore, it inhibits mineralization in vitro [54,55]. This last property finds partial confirmation in the downregulation of ALPL, which is an early and late mineralization gene.

The overall data suggest that both MBG_Sr2%_SG and MBG_Sr2%_SD are able to promote bone formation, stimulating the expression of COLL1A1, SPARC and OPG and a down regulation of RANKL.

Overall this preliminary in vitro biological assessment evidenced the biocompatibility of the produced Sr-containing MBGs and confirmed the anti-inflammatory and pro-osteogenic effect of Sr^2+^ ions [46,54,56,57,58] released by MBGs micro and nanoparticles.

## 4. Conclusions

Mesoporous bioactive glasses incorporating different amounts of strontium were successfully prepared through two different synthesis procedures, a base-catalysed sol-gel and an aerosol-assisted spray-drying method, in the form of nano- and micro-particles, respectively. Besides the size of the final particles, the two synthesis approaches allowed to obtained samples with different specific surface area, pore volume and average pore size.

Release tests carried out in Tris HCl revealed that Sr^2+^ was almost totally released within 7 days with final released concentrations well correlated to the incorporated amount. 

The samples showed an excellent bioactive behaviour, evidenced by the formation of HA deposits after 1 day of soaking in SBF, demonstrating that the partial substitution of calcium with strontium does not significantly affect the surface ion-exchange kinetics. 

Both type of Sr-containing MBGs were biocompatible, showed a reduced pro-inflammatory response and were able to stimulate the expression of pro-osteogenic genes (COLL1A1, SPARC and OPG), confirming the potential of Sr^2+^ as therapeutic element for the stimulation of bone remodelling.

Based on the obtained results, Sr-containing MBGs are very promising candidates for bone regenerative applications as such or in association with drugs as multifunctional carriers in combination with polymeric matrices. 

## Figures and Tables

**Table 1:** Amounts of used salt precursors for MBG_Sr2%_SD and MBG_Sr4%_SD.

Reagent	MBG_Sr2%_SD (g)	MBG_Sr4%_SD (g)
SrCl_2_·6H_2_O	0.32	0.64
Ca(NO_3_)_2_·4H_2_O	1.86	1.57

**Table 2:** Amounts of used salt precursors for MBG_Sr2%_SG and MBG_Sr4%_SG.

Reagent	MBG_Sr2%_SG (g)	MBG_Sr4%_SG (g)
SrCl_2_·6H_2_O	0.84	1.68
Ca(NO_3_)_2_·4H_2_O	4.88	4.13

**Table 3:** Main textural characteristics of Sr-containing MBGs obtained by N_2_ adsorption/desorption analysis.

Parameters	MBG_Sr2%_SG	MBG_Sr4%_SG	MBG_Sr2%_SD	MBG_Sr4%_SD
BET surface area	803 m^2^·g^−1^	551 m^2^·g^−1^	167 m^2^·g^−1^	154 m^2^·g^−1^
Average Pore size	4.8 nm	4.1 nm	8.3 nm	7.8 nm
Pore volume	0.82 cm³·g^−1^	0.45 cm³·g^−1^	0.18 cm³·g^−1^	0.17 cm³·g^−1^

**Table 4:** Sr^2+^ amount incorporated expressed in mol % and ppm, Sr^2+^ released quantity in ppm after 3 h and 72 h of soaking in Tris HCl.

Sample	Sr^2+^ Incorporated mol %	Sr^2+^ Incorporated ppm	Sr^2+^ Released at 3 h (ppm)	Sr^2+^ Released at 72 h (ppm)
MBG_Sr2%_SG	1.0%	2.40	2.19	2.36
MBG_Sr4%_SG	2.0%	7.60	6.68	7.60
MBG_Sr2%_SD	2.0%	7.20	6.30	6.90
MBG_Sr4%_SD	3.7%	13.20	12.1	13.2

**Table 5:** RANKL/OPG ratio at 72 h and 7 days, for MBG_Sr2%_SG, MBG_Sr2%_SD and polystyrene. A significant down regulation of RANKL and an overexpression of OPG, results in a RANKL/OPG ratio in favour of bone remodelling at both time points.

Sample	RANKL/OPG
Polystyrene 72 h	1.000
MBG_Sr2%_SG 72 h	0.181
MBG_Sr2%_SD 72 h	0.230
Polystyrene 7 days	2.291
MBG_Sr2%_SG 7 days	0.563
MBG_Sr2%_SD 7 days	0.275
